# Haemorrhagic Retroperitoneal Paraganglioma: A Report of a Rare Case

**DOI:** 10.7759/cureus.77242

**Published:** 2025-01-10

**Authors:** João Campos-Cunha, João B Martins, Gonçalo Carneiro, Helena Maia

**Affiliations:** 1 Internal Medicine, Centro Hospitalar de Entre Douro e Vouga, Santa Maria da Feira, PRT

**Keywords:** acute abdomen, haemorrhage, neuroendocrine, paraganglioma, treatment

## Abstract

Catecholamine-producing tumours are rare entities that, even though their clinical diagnostic might be a challenge due to the non-specificity of the symptoms, have had a growing incidence due to the continuous improvement of medical imaging examinations and the evolution of molecular genetic testing. On the other hand, they can rarely manifest as serious complications, such as myocardial infarction, stroke, or alveolar haemorrhage.

This paper describes the case of a 77-year-old Caucasian man who presented with acute onset of left upper quadrant abdominal pain. The first abdominal computed tomography (CT) scan showed an active haemorrhage originating from a retroperitoneal paraganglioma. The patient received intravenous fluids and prothrombin complex concentrate for reversal of anticoagulation therapy. A reassessment CT scan performed 12 hours later suggested increased bleeding, and laboratory findings showed a worsening of the anemia, so an angiography was performed which didn't show any evidence of haemorrhage. After a multidisciplinary discussion, it was decided to admit the patient for surveillance and imaging evaluation. Six days later, a second reassessment CT showed signs of haemorrhagic resolution, and the patient was discharged with subsequent follow-up in the outpatient clinic.

The authors highlight this case for being a rare complication of an equally rare neuroendocrine tumour.

## Introduction

Paragangliomas are rare neuroendocrine tumours, originating in the ganglia of the sympathetic or parasympathetic nervous system, which can secrete catecholamines. They are histologically indistinguishable from pheochromocytomas, so the distinction is made through their position, being classified as paragangliomas if detected in an extra-adrenal location or pheochromocytomas if intra-adrenal [1]. Despite no histological differentiation, the distinction between these two groups is important both in the context of genetic testing and also due to the inherent risk of malignancy [2]. These tumours have an estimated incidence of 0.6 cases per 100,000 individuals per year [3], and, just like pheochromocytomas, paraganglioma's diagnostic can be difficult, as symptoms such as palpitations, hyperhidrosis, or headache can also appear in several other more common medical conditions [3].

Paraganglioma's location can be established through anatomical examinations such as computed tomography (CT) and magnetic resonance imaging (MRI) or functional exams such as positron emission tomography and scintigraphy. The treatment of choice in most cases involves surgical resection, preferably by laparoscopic approach [4], combined with preoperative alpha- and beta-adrenergic block to prevent potential intraoperative hypertensive crisis [3].

Even though most of these tumours are sporadic, they can also be associated with genetic mutations, integrating syndromes such as multiple endocrine neoplasia type 2 (MEN2), neurofibromatosis type 1, or von Hippel-Lindau disease [3].

## Case presentation

A 77-year-old man was evaluated in the emergency department for a five-hour history of acute onset of left upper quadrant abdominal pain. He had a history of a retroperitoneal neuroendocrine tumour located between the pancreas and the left renal vessels under investigation, hypertension medicated with three classes of drugs, stage IIIb chronic kidney disease, and atrial fibrillation medicated with rivaroxaban. With regard to the physical examination, the patient was tachycardic but hemodynamically stable, with a distended and soft abdomen, with pain in the left flank but without guarding.

**Figure 1:**
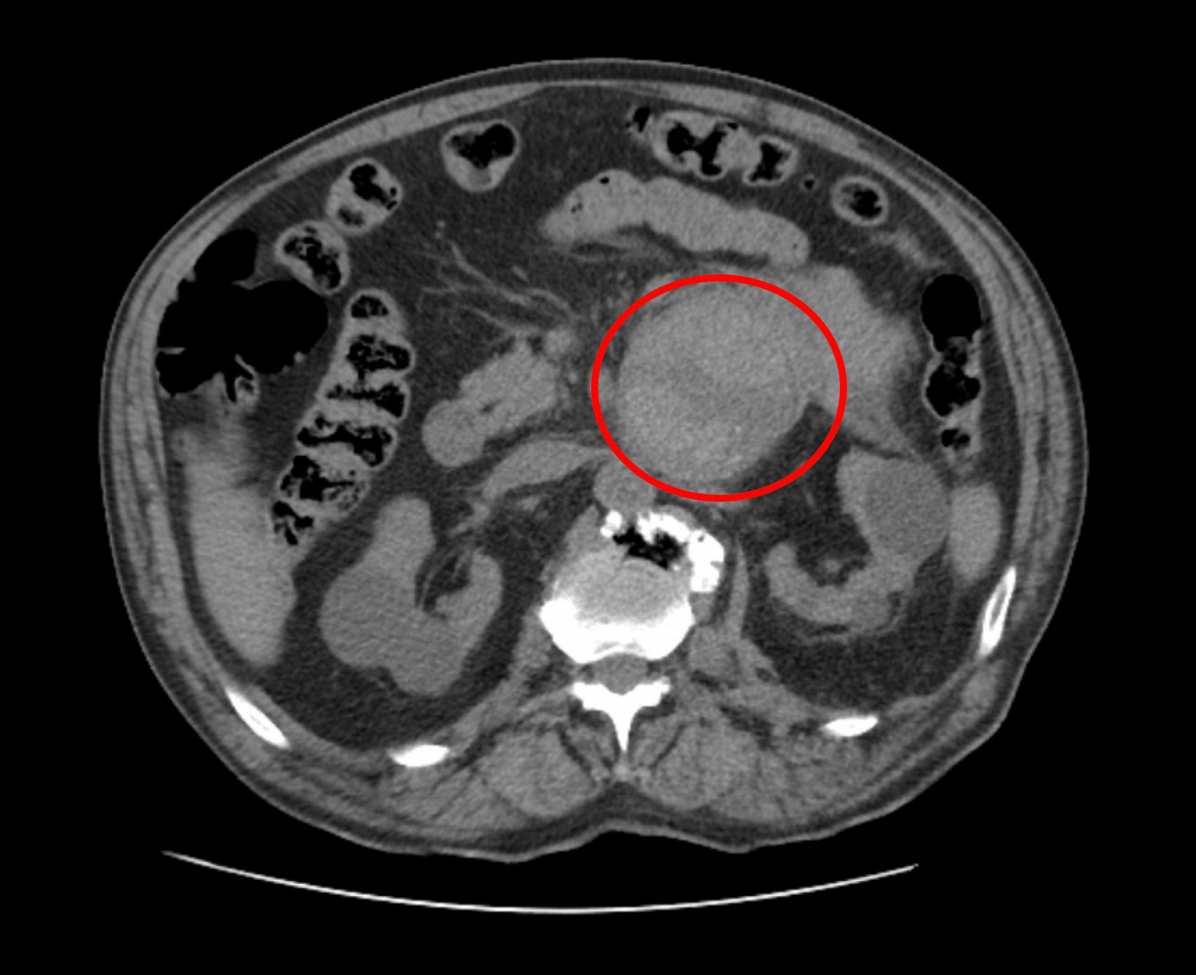
Abdominal computed tomography at admission, without contrast, showing the tumor (circle)

**Figure 2:**
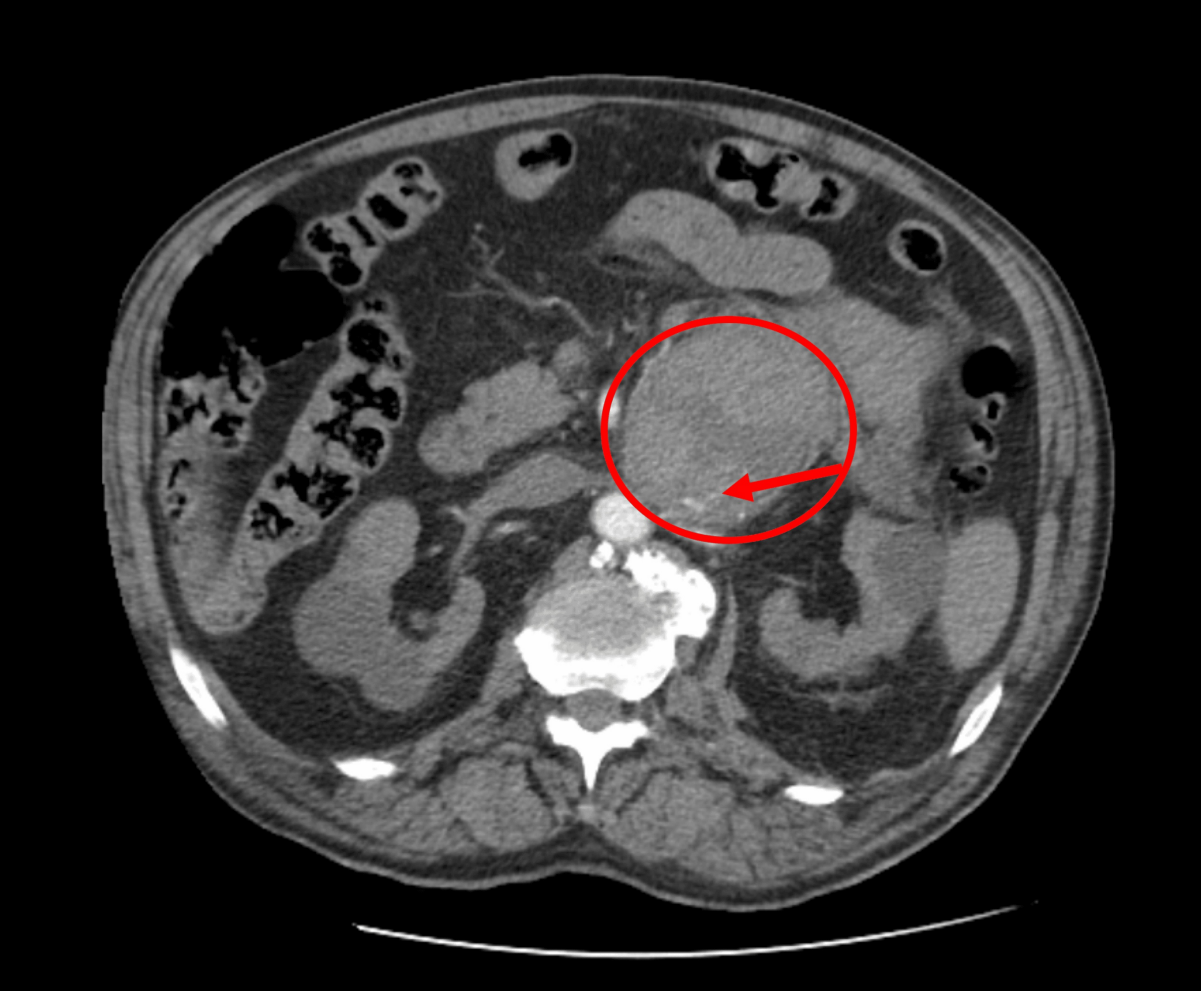
Abdominal computed tomography at admission, in the arterial phase, showing vascular blush suggestive of active bleeding (arrow)

Blood tests showed mild macrocytic anemia (hemoglobin 11.9 g/L, mean glomerular volume 104.6 fL) and worsening of his baseline renal function (creatinine 2.6 mg/dL), as depicted in Table 1. Arterial blood gas analysis (Table 2) indicated metabolic acidemia (pH 7.26, bicarbonate (HCO3-) 8.1 mmol/L), hyperkalemia (potassium 5.8 mmol/L), and hyperlactacidemia (lactate 7.1 mmol/L), which were corrected with fluid therapy and hypokalemic measures. Abdominopelvic CT with contrast raised the suspicion of a venous haemorrhage with a starting point on the tumour (Figure 1 and Figure 2), so prothrombin complex concentrate was administered. After a period of surveillance of 12 hours, a reassessment CT scan was performed, which showed increased bleeding and suggested the presence of active arterial haemorrhage. In the analytical reassessment, there was a worsening of the anemia (hemoglobin 10.1 g/L).

**Table 1:** Analytical parameters at admission and reassessment

Analytical parameters	Reference range/unit	Value at admission	Value at reassessment
Hemoglobin	12.0-16.0 g/dL	11.9	10.1
Hematocrit	40-50%	38.4	32.3
Leucocytes	4.0-11.0 × 10^9^/L	8.4	10.7
Platelets	150-400 × 10^9^/L	283	225
Prothrombin time	10-13 seconds	11.9	11.6
Partial thromboplastin time	25-35 seconds	31.5	33.5
Urea	18-55 mg/dL	92	97
Creatinine	0.7-1.3 mg/dL	2.6	2.6
Aspartate transaminase	5-34 U/L	29	-
Alanine transaminase	0-55 U/L	16	-
Gamma-glutamyl transferase	<38 U/L	73	-
Alkaline phosphatase	40-150 U/L	85	-
Total bilirubin	0.20-1.20 mg/dL	0.35	-
Amylase	28-100 U/L	201	-
Lipase	8-78 U/L	73	-
Lactate dehydrogenase	125-220 U/L	224	271
C-reactive protein	<5.0 mg/L	5.1	13.9

**Table 2:** Arterial blood gas values at admission PaCO2: partial pressure of carbon dioxide; PaO2: partial pressure of oxygen; HCO3-: bicarbonate; SaO2: oxygen saturation

Analytical parameters	Reference range/unit	Value at admission
pH	7.35-7.45	7.26
PaCO2	35-45 mmHg	18
PaO2	80-100 mmHg	112
HCO3-	22-26 mEQ/L	8.1
Potassium	3.5-5.0 mg/dl	5.8
Lactate	<2.0 mmol/L	7.1
SaO2	93-98%	99

The patient was evaluated by interventional radiology, who performed an angiography that didn't show any evidence of haemorrhage. Due to the absence of active bleeding and the hemodynamic stability of the patient, it was considered that there was no need for urgent embolization.

**Figure 3:**
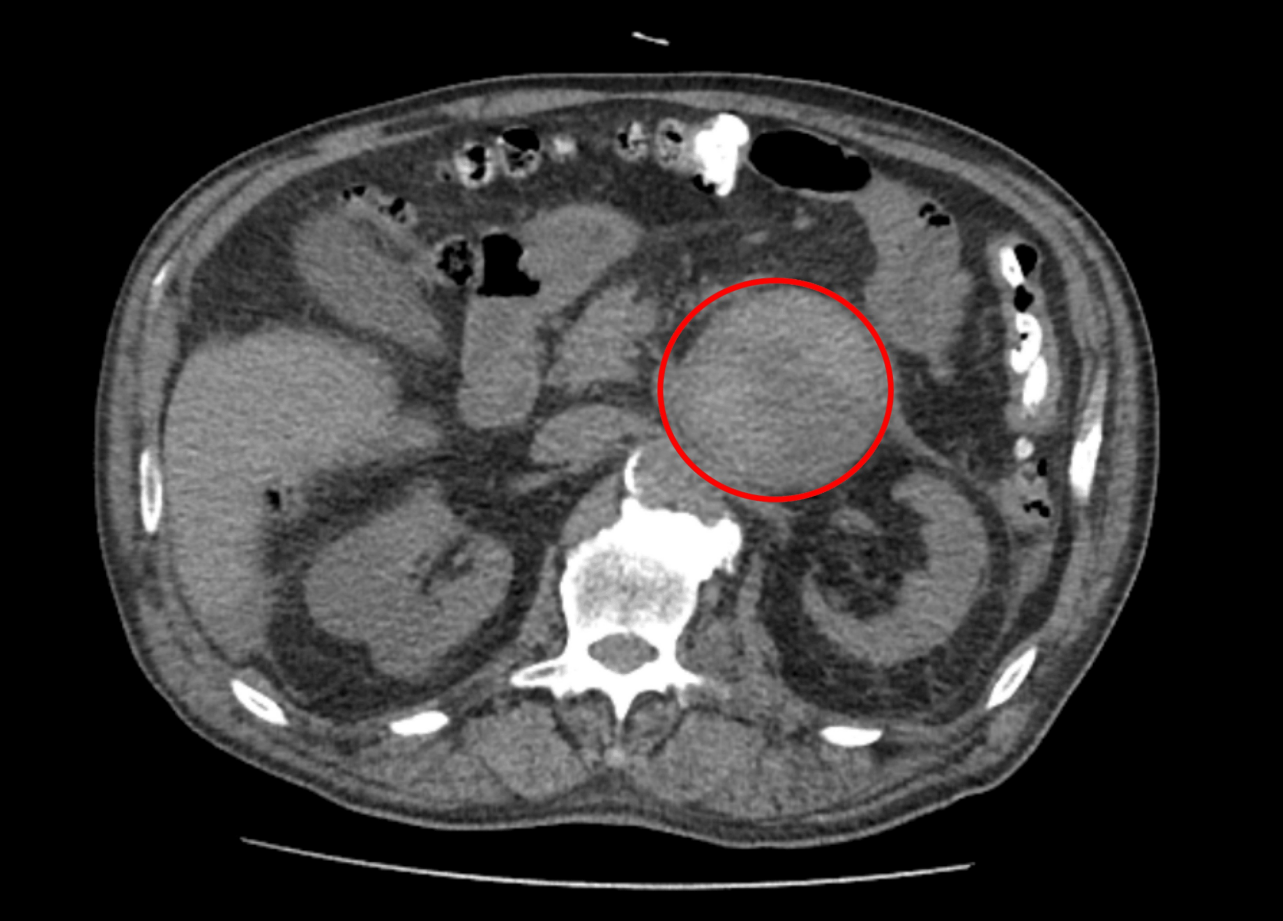
Computed tomography at discharge (without contrast): lesion with slight reduction in size and more homogeneous density, suggestive of haemorrhage in the resolution phase

The patient was admitted for study and surveillance, with a functional study demonstrating the production of catecholamines. He had a favourable outcome, with haemorrhagic resolution on a second reassessment CT performed six days after the first one (Figure 3).

## Discussion

The increasing availability of imaging tests such as CT or MRI has led to a progressive increase in the diagnosis of this type of tumours, with approximately 40% of paragangliomas or pheochromocytomas being diagnosed as incidentalomas [5].

Ideally, all patients with these diagnoses should be screened for associated genetic mutations, since they usually have an autosomal dominant transmission, which means that direct relatives of these patients have a 50% chance of being carriers. Therefore, its early identification can lead to a better analytical and radiological follow-up, for both patients and their relatives. Patients with these genetic syndromes are, on average, diagnosed about 15 years earlier than sporadic cases, in addition to having more frequent tumours in atypical locations such as the chest or bladder [3]. This screening is even more important considering that up to half of paragangliomas are associated with these mutations [3].

Although in this specific patient the genetic study was not carried out while hospitalized, we are probably facing a sporadic case, not only because it is a single tumour but also due to the patient's age, since the probability of having a genetic syndrome is practically null when the age at diagnosis is above 60 years old [5]. On the other hand, it is important to note that the risk of malignancy is higher in sporadic tumours and that sympathetic paragangliomas have a higher risk of malignancy than pheochromocytomas, which can also occur in up to 50% of cases [6]. However, even patients with metastatic disease have a fairly long life, with an estimated average survival of 34 years from diagnosis [7].

The bleeding mechanisms of these tumours are not fully understood, with some theories speculating pathophysiological processes such as fast tumour expansion outgrowing its vascular supplementation, increased intracapsular pressure due to trauma, or side effects of antihypertensive drugs [8]. From the review of the medical literature, we could see that haemorrhages of retroperitoneal paragangliomas presenting as acute abdomen are extremely rare phenomena. After conducting a search in PubMed, Web of Science, and OVID, Yang et al. [9] described only two cases with these characteristics.

It should be noted that, unlike pheochromocytomas, paragangliomas can have several arterial vessels coming from adjacent arteries, so their removal can lead to bleeding complications [5]. On the other hand, medical literature suggests that, in these cases, urgent surgery should be avoided, especially when the haemorrhage is contained, since the decision of having delayed surgery is associated with a lower rate of intraoperative complications and a reduction of the mortality rate [10]. In the present case, it was with this in mind that the decision of hospitalization for surveillance was taken instead of urgent surgery.

After haemorrhagic resolution, the patient was not operated on because he refused to, being discharged to the outpatient clinic. He had a follow-up for about a year, maintaining the will of avoiding surgery and signing a term of responsibility.

## Conclusions

The main objective of this case was to report a rare but potentially serious intercurrence of a neuroendocrine tumour. Although rare, the incidence of these tumours has been increasing in recent years, thanks to the advent of progressively more developed medical imaging modalities. Its treatment is usually surgical, and patients as well as their direct relatives should be offered integration into a genetic screening program and long-term follow-up.
